# Cdk3-promoted epithelial-mesenchymal transition through activating AP-1 is involved in colorectal cancer metastasis

**DOI:** 10.18632/oncotarget.6875

**Published:** 2016-01-09

**Authors:** Jinping Lu, Zhen Lin Zhang, Damao Huang, Na Tang, Yuejin Li, Zhengke Peng, Chengrong Lu, Zigang Dong, Faqing Tang

**Affiliations:** ^1^ Clinical Laboratory and Medical Research Center, Zhuhai Hospital of Jinan University, Zhuhai People's Hospital, Zhuhai, P.R. China; ^2^ Clinical Laboratory, Xiangya Hospital of Central South University, Changsha, P.R. China; ^3^ Institution of Pathogenic Biology, Medical College, University of South China, Hengyang, P.R. China; ^4^ Hormel Institute, University of Minnesota, Austin, Minnesota, USA

**Keywords:** Cdk3, colorectal cancer, AP-1, epithelial-mesenchymal transition, metastasis

## Abstract

Cyclin dependent kinase-3 (Cdk3) is a positive regulator of the G1 mammalian cell cycle phase. Cdk3 is involved in cancer progression, but very little is known about its mechanism in cancer development and progression. Herein, we found that Cdk3 increased colorectal cancer metastasis through promoting epithelial-mesenchymal transition (EMT) shift. Cdk3 was found to highly express in metastatic cancer and induce cell motility and invasion. Cdk3 was shown to phosphorylate c-Jun at Ser 63 and Ser 73 *in vitro* and *ex vivo*. Cdk3-phosphorylated c-Jun at Ser 63 and Ser 73 resulted in an increased AP-1 activity. Ectopic expression of Cdk3 promoted colorectal cancer from epithelial to mesenchymal transition conjugating AP-1 activation, while AP-1 inhibition dramatically decreased Cdk3-increased EMT shift. These results showed that the Cdk3/c-Jun signaling axis mediating epithelial-mesenchymal transition plays an important role in colorectal cancer metastasis.

## INTRODUCTION

Colon cancer is one of the most common malignancies worldwide, with about 1.2 million new cases and 608,700 deaths every year [1, 2]. In the United States, an estimated 40,000 new cases of rectal cancer occurred, and an estimated 50,310 people died from rectal and colon cancer combined in 2014 [3]. In Eastern Asia, rectal cancer occurs in more than 16 per 100,000 individuals per year and accounts for more than 168,000 death [3, 4]. Colorectal cancer is one of the major death causes, being the third most common diagnosed cancer in men and the second in women [5], and metastasis is the main factor of colorectal cancer death [6, 7]. Synchronous distant metastases is already observed in about 30% of colorectal cancer patients, and at least a further third develops metachronous metastases despite primary treatment with curative intention [6, 8]. Therefore, development of distant metastases is the most crucial and lethal event, critically limiting therapy options [9, 10].

Metastasis is a complex process, going along with strong morphological and functional changes of tumor cells [11]. One of the earliest steps of metastasis is to escape from the primary tumor to invade surrounding blood vessels [12]. Epithelial-to-mesenchymal transition (EMT) is critical for tumor cell invasion and dissemination. Many factors are involved in development and progression of colorectal cancer metastasis, but its mechanism is still not clear. The transcription factor Forkhead box M1 (FOXM1) overexpression was significantly associated with lymph node metastasis, its downregulation inhibited cell migration and invasion *in vitro*. In mechanism, its downexpression reversed EMT phenotype by up-regulating E-cadherin, reduction vimentin and snail expressions [13]. SOX9 bound to and activated S100P promoter. Knockdown of SOX9 expression decreased S100P expression, resulting in the reduced invasiveness and metastasis of colon cancer cells by inhibiting EMT shift [14].

Cyclin-dependent kinase (Cdk3) is a kinase that enhances progression through G1 into S phase in the mammalian cell cycle [15–17]. Cdk3 activation appears early in G1-phase [18] and peaks at mid G1 [15], and is required for entry into S-phase [16]. During G1/S transition, Cdk3 binds to E2F-1, E2F-2, or E2F-3 through DP-1 and enhances their transcriptional activities [19], thus playing an essential role at the G1/S transition. More importantly, Cdk3 highly expresses in various cancer [20–22], and its expression is associated with the degree of infiltration, lymph node metastasis and clinical staging [21, 22]. As a positive regulator of G1, Cdk3 is involved in malignant cell transformation [15, 23]. The previous works showed that Cdk3 phosphorylates c-Jun at Ser63/73 and increases AP-1 activity [24]. AP-1 activation induces EMT shift, and participates in cancer metastasis [25, 26]. We speculated that Cdk3 may mediate AP-1 activation, promote EMT shift, and promote colorectal cancer metastasis. In the present study, we found that Cdk3 is highly expressed in metastatic colorectal cancer. It was confirmed that Cdk3 ectopic expression increases cell motility and invasion *in vitro*, enhances colorectal cancer metastasis *ex vivo*. Additionally, Cdk3 was also found to induce colorectal cancer cell from epithelial to mesenchymal transition through activating AP-1. Our data showed that Cdk3 is involved in colorectal cell metastasis.

## RESULTS

### Expression of Cdk3 in normal colorectal tissues, colorectal cancer and metastatic cancer tissues

**Figure 1:**
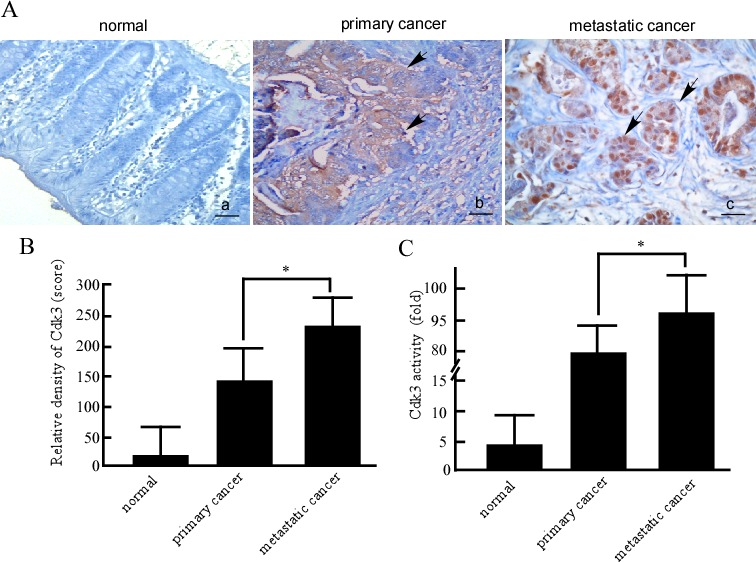
Cdk3 expression and activity in normal colorectal, primary cancer and metastatic cancer tissues **A.** The formalin-fixed and paraffin-embedded tissue sections of normal colorectal tissues, colorectal primary cancer, and metastatic cancer were stained using a standard immunohistochemical technique. Cdk3 was stained with Cdk3 antibody in the tissues samples. Arrows, positive cells. Original magnification, ×200. Scale bar, 10 μm. **B.** Evaluation of Cdk3 staining. Each case was rated according to a score that added a scale of intensity of staining to nucleus and cytoplasm. **C.** Cdk3 activity was detected in normal colorectal, primary and metastatic cancer tissues using *vitro* kinase assay as described in Material and methods. Three individual experiments and presented as mean ± SD. Statistical analysis was done using SPSS (version 18.0), a difference of *P* < 0.05 was considered statistically significant.*, represented *P* < 0.05.

To know the relationship of Cdk3 and colorectal cancer metastasis, Cdk3 expression was detected using immunohistochemistry in formalin-fixed and paraffin-embedded archival clinical tissues, including 52 cases of normal colorectal tissue, 87 cases of primary colorectal cancer, and 49 cases of metastatic colorectal cancer. The immunohistochemistry results revealed positive Cdk3 signals showing brown-yellow granules in the cytoplasm and nucleus (Figure 1A–b, c). Cdk3 staining scores were evaluated according to staining intensity and area in both nuclear and cytoplasm. The results showed that Cdk3 scores were stronger in metastatic cancer than that in primary cancer, and primary cancer being stronger than in normal colorectal tissues (Figure 1B). Furthermore, as shown in Table 1, the percentages of Cdk3 positive staining in normal, primary cancer and metastatic cancer tissues were respectively 11.5%, 21.8% and 83.7 %. Cdk3 protein expression was significantly increased in metastatic cancer compared to primary cancer (*P* < 0.05). Additionally, the correlation of CDK3 expression with clinical features and prognosis of colorectal cancer patients was analyzed, Cdk3 expression is related with TNM grade (Supplemental Table, *P* < 0.05). As a kinase, Cdk3 activity mainly contributes to Cdk3 function. Further, we detected Cdk3 activity in normal colorectal tissue, primary and metastatic cancer using *in vitro* kinase assay. The results showed that Cdk3 activity is the highest in metastatic cancer tissues, and being higher in metastatic cancer than that in primary one (Figure 1C, *P* < 0.05). This suggests that Cdk3 expression may be related to colon cancer progression.

**Table:** Cdk3 expression in normal colon tissue, colon cancer and metastatic cancer

Group	*n*	−	+	%	*P*
normal	52	46	6	11.5	
primary cancer	87	67	19	21.8	0.027
metastatic cancer	49	8	41	83.7	0.001

### Cdk3 increases motility and invasion of colorectal cancer cells

**Figure 2:**
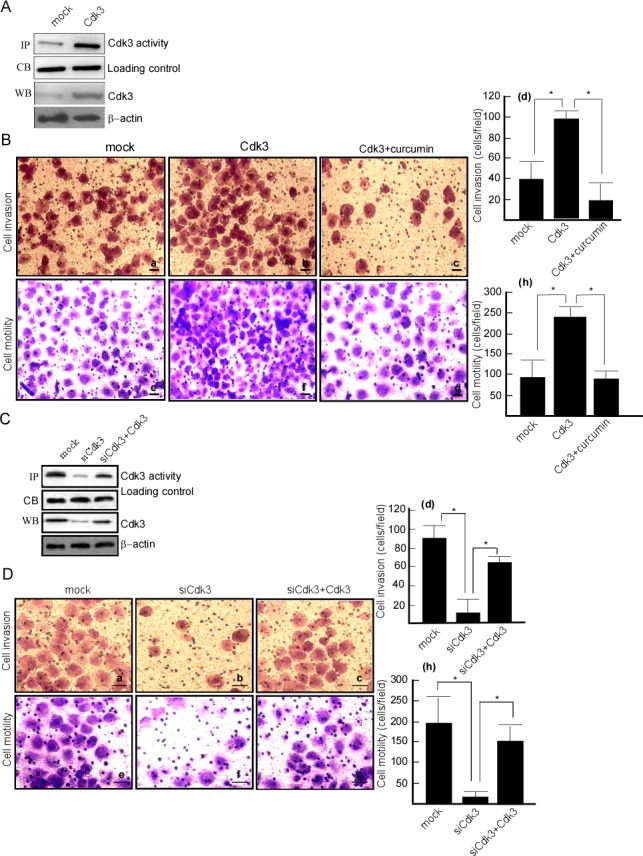
Cdk3 increases the motility and invasion of colorectal cancer cells **A.** Cdk3 expression was detected in HT29-Cdk3 and HT29-mock using Western-blotting. Cdk3 activity was detected using the immunoprecipiptation and *in vitro* kinase assay. **B.** Matrigel-coated Boyden chamber was used to measure the invasion of HT29-mock (a), HT29-Cdk3 (b), and HT29-Cdk3 with curcumin treatment (c). Random fields of view were counted to determine the number of invading cells (d). The uncoated Boyden chamber was used to determine these cells motility (e, f, g) and random fields were counted to determine the number of motility cells (h). **C.** Cdk3 expression and activity were detected in SW620-simock, SW620-siCdk3 and SW620-siCdk3 with Cdk3 transfection. **D.** The invasion of SW620-simock (a), SW620-siCdk3 (b) and SW620-siCdk3 with Cdk3 transfection was detected (c). The invasion cells were counted (d). The motility of SW620-simock (a), SW620-siCdk3 (b) and SW620-siCdk3 with Cdk3 transfection was detected (e, f, g), and the motility cells were counted (h). Original magnification, ×400. Scale bar = 20μm. The cells were counted in three individual experiments and presented as mean ± SD. *, represented *P* < 0.05. Coommassie blue staining and β-actin served as a loading control. IP, immunoprecipiptation assay; CB, Coommassie blue staining; WB, Western-blotting analysis. *, represented *P* < 0.05.

The above suggests that Cdk3 may be involved in colorectal cancer metastasis. Next step is to investigate whether Cdk3 increases the motility and invasion of cancer cell. HT29 cell, a colon cancer cell line with a low metastatic ability was transfected with pRcCMV-HA-Cdk3 (HA-Cdk3), and the stable expressed cell line, HT29-Cdk3 was obtained by selection for G418 resistance. Its motility and invasion was detected using Boden chamber invasion assay *in vitro*. HT29 cell had a low expression (Figure 2A, left lane in 3^rd^ panel) and activity of Cdk3 (Figure 2A, left lane in 1^st^ panel), and the expression (Figure 2A, right lane in 3^rd^ panel) and activity (Figure 2A, right lane in 1^st^ panel) of Cdk3 dramatically increased when being transfected with Cdk3. Boden chamber assay showed that HT29 cell had a low motility (Figure 2B–d) and invasion ability (Figure 2B-a), and the motility (Figure 2B-e) and invasion (Figure 2B-b) ability significantly increased after being transfected Cdk3 (Figure 2B-(d, h), *P* < 0.05). These results indicate that Cdk3 increases the motility and invasion of colorectal cancer cell. To further observe AP-1's effect on Cdk3-increasing motility and invasion, AP-1 activity was inhibited in HT29-Cdk3 using AP-1 inhibitor curcumin, the motility and invasion were measured. After curcumin treatment, the motility (Figure 2B-g) and invasion (Figure 2B-c) dramatically decreased (Figure 2B-(d, h), *P* < 0.05). AP-1 plays an important role in Cdk3-increasing cell motility and invasion.

To eliminate cell-line specific phenomenon, we also used colorectal cancer cell lines HCT116 and SW480 to further investigate, HCT116 as high metastatic ability cell, and SW480 as low metastatic cell, and got the similarity results. Cdk3 increased SW480 motility and invasion (Supplemental Figure 1A, 1B), siCdk3 decreased HCT116 motility and invasion (Supplemental Figure 1C, 1D).

### Cdk3 knockdown decreases motility and invasion of colorectal cancer cells

To confirm Cdk3's role in colorectal cancer metastasis, the motility and invasion of colorectal cancer cells was observed when Cdk3 knockdown. SW620 cell, a colon cancer cell line with a high metastatic ability was transfected with siRNA-Cdk3 (siCdk3). SW620-siCdk3 was obtained by selection for G418 resistance. SW620 cell had a high Cdk3 expression(Figure 2C, left lane in 3^rd^ panel) and activity (Figure 2C, left lane in 1^st^ panel), and the expression (Figure 2C, middle lane in 3^rd^ panel) and activity (Figure 2C, middle lane in 1^st^ panel) of Cdk3 dramatically decreased when being transfected siCdk3. Boden chamber assay showed that SW620 cell had a high motility (Figure 2D-e) and invasion ability (Figure 2D-a), and the motility (Figure 2D-f) and invasion (Figure 2D-b) ability significantly decreased in the transfect with siCdk3 (Figure 2D-d,h *P* < 0.05). To further confirm Cdk3's role in cell motility and invasion, a rescue experiment for Cdk3 knockdown was conducted, SW620-siCdk3 were transfected with pRcCMV-Cdk3. The results showed that cell motility (Figure 2D-g) and invasion (Figure 2D-c) were increased after being transfected with Cdk3 (Figure 2D-d,h *P* < 0.05). SW620-siCdk3 regained motility and invasion ability after Cdk3 rescue. These data indicate that Cdk3 may play an important role in colorectal cancer metastasis.

### Cdk3 binds to and co-localizes with c-Jun

**Figure 3:**
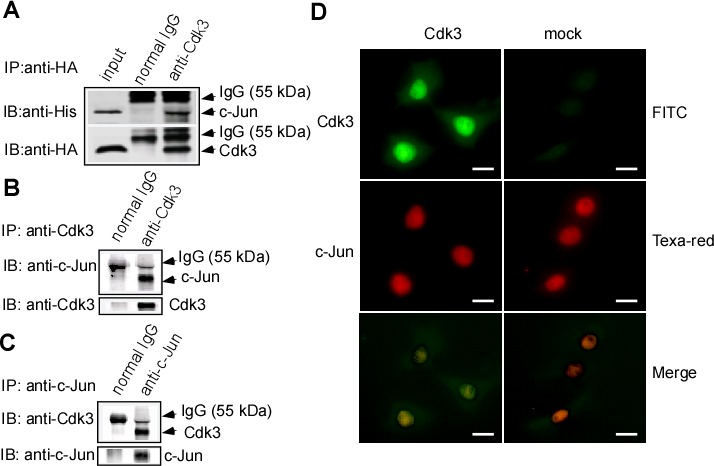
Binding of Cdk3 and c-Jun protein **A.** Cdk3 binds to c-Jun in exogenous expression. pRcCMV-HA—Cdk3 and pHis6-tagged c-Jun plasmids were transiently co-transfected into HCoEPiC cells. Cdk3 in His-tagged protein was detected by Western-blotting using anti-HA, and c-Jun was probed using anti-His. Inputs are representative of whole cell lysates. Precipitation with normal IgG served as a negative control. **B.** HT29-Cdk3 cells were used to investigate the binding of Cdk3 and c-Jun in endogenesis expression. Cdk3 in HT29-Cdk3 or HT29-mock cells was immunoprecipitated using anti-Cdk3, and the binding with c-Jun was visualized by Western-blotting. **C.** c-Jun in HT29-Cdk3 or HT29-mock cells was immunoprecipitated using c-Jun antibody. Cdk3 and c-Jun proteins were visualized by Western-blotting analysis. **D.** Cdk3 co-localizes and binds to c-Jun *ex vivo*. HT29-Cdk3 and HT29-mock cells were fixed with paraformaldehyde, stained for Cdk3 (green) and c-Jun (red), and then visualized by immunofluorescence microscopy. The localization and binding of Cdk3 and c-Jun are indicated. Original magnification, ×1000. Scale bar, 50 μm. IP: immunoprecipitation; WB: Western-blotting.

Our previous works showed that Cdk3 activates AP-1 through binding to c-Jun in its mediating cell transformation. To confirm whether Cdk3-activating AP-1 exists in cancer metastasis, we co-transfected pHis6-tagged c-Jun (His-c-Jun) and pRcCMV-HA-Cdk3 (HA-Cdk3) into HEK 293 cells. These transfects were used for immunoprecipitation with HA antibody, and the immunocomplex was detected by Western-blotting with the His antibody or HA antibody. His-c-Jun was detectable in the immunocomplex (Figure 3A, lane 3 in upper panel), simultaneously, Cdk3 was also detected in the immunoprecipitation (Figure 3A, lane 3 in down panel). These results show that Cdk3 could bind to c-Jun.

Because endogenous Cdk3 levels are extremely low in most cell types [17], Cdk3- stable expressed cell line, HT29-Cdk3 cell was used in the next experiments. Cdk3 was immunoprecipitated from HT29-Cdk3 with Cdk3 antibody, and c-Jun and Cdk3 proteins in the immunocomplex were analyzed by Western-blotting. c-Jun was detectable in anti-Cdk3 precipitates (Figure 3B, lane 2 in upper panel). Furthermore, c-Jun was immunoprecipitated with c-Jun antibody, Cdk3 was detectable in the c-Jun immunocomplex (Figure 3C, lane 2 in upper panel). These results indicate that endogenous Cdk3 binds with c-Jun in HT29-Cdk3 cell.

Next, we determined whether Cdk3 co-localizes with c-Jun. Cdk3 and mock stably-transfected cells were stained with Texas Red to detect c-Jun and fluorescein- isothiocyanate (FITC) for detecting Cdk3 by immunofluorescence. The results indicate that Cdk3 co-localizes with c-Jun in the nucleus (Figure 3D, left 3^rd^ panel).

### Cdk3 phosphorylates c-Jun and increases AP-1 activity

**Figure 4:**
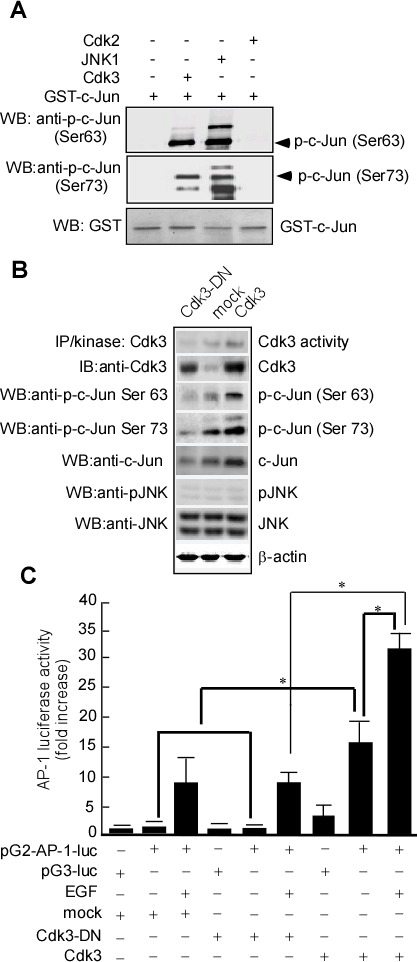
Cdk3 activates AP-1 through phosphorylating c-Jun **A.** *In vitro* kinase assay of Cdk3 to c-Jun. GST-c-Jun protein served as substrate for active Cdk3. Reactive products were subjected to Western-blotting using phosphorylation antibody against c-Jun Ser 63 or Ser 73. GST with Western-blotting staining served as a loading control. The reaction of GST-c-Jun and active JNK1 served as a positive control. **B.** pRcCMV-HA—Cdk3 and pHis6-tagged c-Jun plasmids were transiently transfected into HEK293 cells. Cdk3 activity was detected using immunoprecipitation and *in vitro* kinase assay. Cdk3, c-Jun, phosphorylated c-Jun at Ser 63 or 73, JNK, and phosphorylated-JNK expressions were detected by Western-blotting. β-actin served as the loading control. **C.** HT29-Cdk3, HT29-Cdk3-DN and HT29-mock cells were transfected with the pG2-AP-luciferase promoter (pG2-AP-1-luc). The cells transfected with the pG3-luciferase promoter (pG3-luc) serviced as vector control. Luciferase activity in the transfected cell was measured using the Luminoskan Ascent plate reader. Three individual experiments and presented as mean ± SD. *, represented *P* < 0.05. IP, immunoprecipitation. WB, Western-blotting. p-c-Jun, phosphorylated c-Jun.

To determine whether Cdk3 phosphorylates c-Jun, an *in vitro* kinase assay was conducted using active Cdk3 as the kinase and a GST-c-Jun fusion protein as the substrate. c-Jun phosphorylated by Cdk3 was determined by Western-blotting analysis using specific phosphorylated antibody against c-Jun Ser63 or Ser73. Active JNK1 and GST-c-Jun served as a positive control (Figure 4A, lane 3 in 1^st^ and 2^nd^ panels). Results showed that Cdk3 could phosphorylate c-Jun at Ser63 (Figure 4A, lane 2 in 1^st^ panel) and Ser73 (Figure 4A, lane 2 in 2^nd^ panel). To further confirm Cdk3 phosphorylating c-Jun, pHis6-tagged c-Jun and pRcCMV-HA-Cdk3, pRcCMV-HA-Cdk3-DN (Dominant negative mutant) or pRcCMV-HA (mock) were transiently transfected into HEK 293 cells. Forty hours after transfection, Cdk3 protein expression was detected by Western-blotting analysis using specific antibodies. The phosphorylation of c-Jun at Ser 63 (Figure 4B, lane 3 in 3^rd^ panel) and c-Jun at Ser73 (Figure 4B, lane 3 in 4^th^ panel) could be detected in the cells transfected with c-Jun and Cdk3. No signal was detected in the cells transfected with c-Jun and Cdk3-DN (Figure 4B, lane 1 in 3^rd^ and 4^th^ panels), and it is weak in the cells transfected with c-Jun or mock (Figure 4B, lane 2 in 3^rd^ and 4^th^ panels) when compared with the group with c-Jun and Cdk3. These results show that Cdk3 phosphorylates c-Jun in *ex vivo*.

A persistent alteration in AP-1 activity can result in enhanced cancer cell metastasis [27, 28]. c-Jun is a major component of AP-1 [29], phosphorylated c-Jun plays an important role in AP-1 activation [24], and AP-1 activation mediated by c-Jun phosphorylation is involved in cell invasion [30, 31]. To confirm whether c-Jun phosphorylation mediated by Cdk3 is responsible for the AP-1 activation, we next transfected AP-1 luciferase reporter plasmid (pG2-AP-1-luc) into HT29-Cdk3, HT29-Cdk3-DN, and HT29-mock cells, AP-1 activity was detected using luciferase reporter assay. The results showed that AP-1 activity in Cdk3 stably-transfected cells with EGF treatment was higher than that without EGF (Figure 4C lanes 8 *vs*. lane 9; * *P* < 0.05), and EGF-induced AP-1 activation response in Cdk3 stably-transfected cells was much greater than the response of the Cdk3-DN stably-transfected cells (Figure 4C, lane 6 *vs*. lane 9; * *P* < 0.05). Without EGF treatment, AP-1 activity was also higher in Cdk3 transfect than that in the mock and Cdk3-DN (Figure 4C, lanes 2, 5 *vs*. lane 8; * *P* < 0.05). Overall, these results imply that Cdk3 is responsible for AP-1 activation.

### Cdk3 promotes colon cancer metastasis *ex vivo*

**Figure 5:**
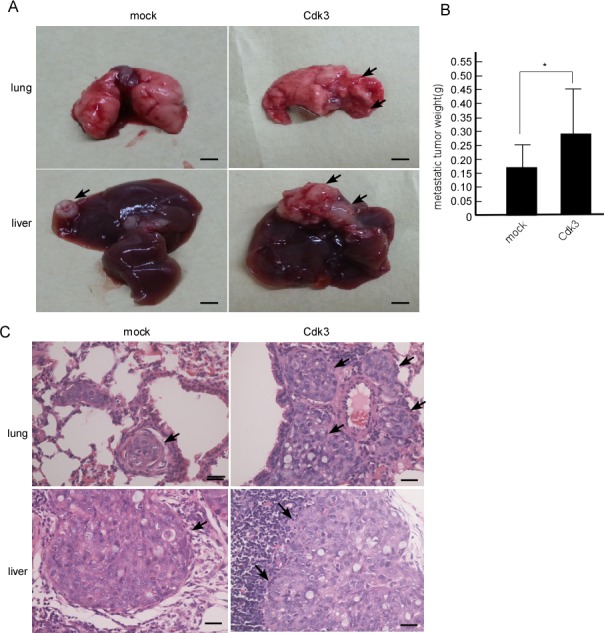
Cdk3 enhances the metastasis of colorectal cancer cell *ex vivo* **A.** 20 nude mice were randomly divided into two groups with 10 mice per group. One group was injected with HT29-Cdk3 cells in Matrigel through the tail vein, and the other group was injected with HT29-mock. After 60 days, the nude mice were sacrificed, and the metastatic tumors were observed. Arrows, metastatic node. **B.** the metastatic tumors from the lung and live were weighed. Scale bar = 0.5 cm (*, *p* < 0.05). **C.** The metastatic tumors were subjected to paraffin embedding and section. Paraffin sections of the metastatic tumors were stained with hematoxylin and eosin. Arrows, metastatic foci. Original magnification, ×400. Scale bar = 20 μm. *, represented *P* < 0.05.

Cdk3-medaited metastasis was confirmed in nude mice, Cdk3 stable-express cell line, HT29-Cdk3 cell line was used in this study. HT29-Cdk3 cells were mixed with Matrigel, and then injected into the tail veins of BABL/c nude mice, HT29-mock served as the control. After 60 days, these mice were sacrificed, and the lung, liver and lymph nodes were observed. To compare with the mock, metastasis of HT29-Cdk3 to the lung and liver significantly increased (Figure 5A), the metastasis to lymph nodes was not observed. The weight of metastatic tumors was increased in HT29-Cdk3 when compared with the mock (Figure 5B, *P* < 0.05). Under microscopy, the metastatic foci in the liver and lung were bigger in HT29-Cdk3 than that in the mock (Figure 5C). These results indicate that Cdk3 enhances colorectal cancer metastasis.

### Exogenous expression of Cdk3 induces EMT-like cellular marker alteration in colorectal cancer cells

**Figure 6:**
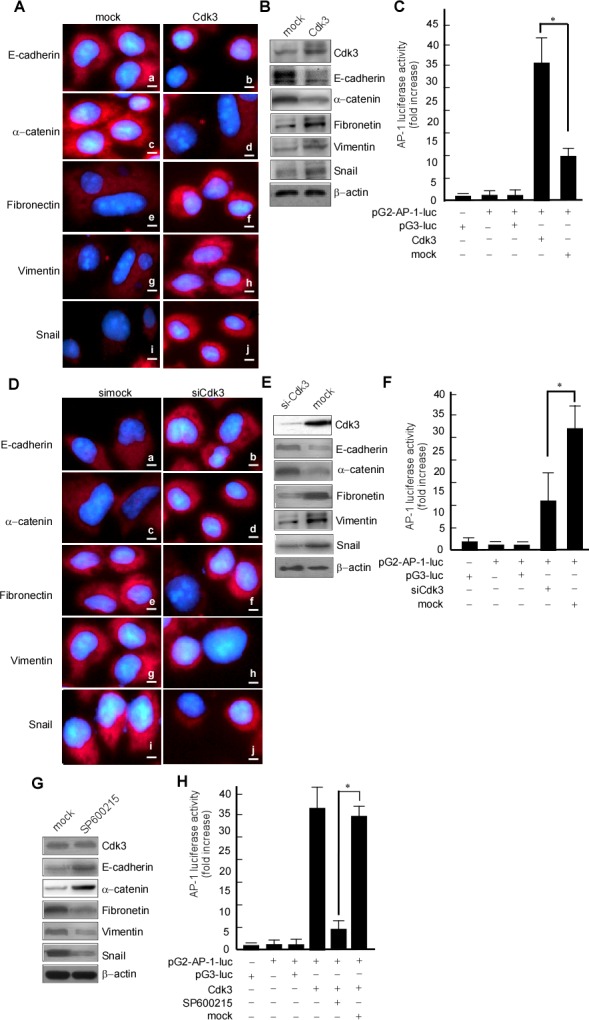
Cdk3 induces EMT-like cellular marker alterations HT29-Cdk3 and HT29-mock cells were used to detect the expression of EMT markers. **A.** E-cadherin (a, b), α-catenin (c, d), fibronectin (e, f), vimentin (g, h), and snail (I, j) were detected in HT29-Cdk3 and HT29-mock cells using immunofluorescence. Magnification, ×1000, scale bar = 50μm. **B.** E-cadherin, α-catenin, fibronectin, vimentin, and snail were detected in HT29-Cdk3 and HT29-mock cells using Western-blotting. **C.** AP-1 activity was detected in HT29-Cdk3 and HT29-mock cells as described in Materials and Methods. Three individual experiments and presented as mean ± SD. **D.** SW620-siCdk3 and SW620-simock cells were used to detect the expression of EMT markers. E-cadherin (a, b), α-catenin (c, d), fibronectin (e, f), vimentin (g, h), and snail (I, j) were detected in SW620-siCdk3 or SW620-simock cells using immunofluorescence. Magnification, ×1000, scale bar = 50μm. **E.** E-cadherin, α-catenin, fibronectin, vimentin, and snail in SW620-siCdk3 and SW620-simock cells were detected using Western-blotting. **F.** AP-1 activity was detected in SW620-siCdk3 and SW620-simock cells as described in Materials and Methods. HT29-Cdk3 cells were treated with curcumin, and E-cadherin, α-catenin, fibronectin, vimentin, and snail were detected using Western-blotting (G), AP-1 activity was detected using luciferase activity assay (H). Three individual experiments and presented as mean ± SD. *, represented *P* < 0.05.

The above results showed that Cdk3 can promote migratory/invasive properties of colorectal cancer. Since the enhanced migratory/invasive ability of epithelial cells is often caused by EMT, we analyzed a panel of representative epithelial and mesenchymal markers to determine whether this process occurs in Cdk3 mediating colorectal cancer metastasis. The results showed that Cdk3 ectopic expression caused an EMT-like marker shift, including downregulation of epithelial markers E-cadherin (Figure 6A-a) and α-catenin (Figure 6A-c), and upregulation of mesenchymal markers fibronectin (Figure 6A-e) and EMT-associated transcription factor snail (Figure 6A-i), vimentin (Figure 6A-g). Western-blotting analysis further revealed that E-cadherin (Figure 6B, 2^nd^ panel) and α-catenin (Figure 6B, 3^rd^ panel) expression dramatically decreased (Figure 6B), while the levels of fibronectin (Figure 6B, 4^th^ panel), vimentin (Figure 6B, 5^th^ panel) and snail (Figure 6B, 6^th^ panel) increased in Cdk3-expressing cells. Simultaneously, AP-1 activity was detected in HT29-Cdk3 and HT29-mock cells. The data showed that AP-1 activity was significantly higher in HT29-Cdk3 than in HT-mock (Figure 6C, lane 4 *vs*. 5, * *P* < 0.05). These results thus demonstrated that Cdk3 induces EMT-like molecular alterations with AP-1 activation in colorectal cancer.

To further investigate whether endogenous Cdk3 contributes to EMT phenomenon, we observed whether colorectal cancer cells lacking endogenous Cdk3 expression demonstrate any EMT-like cellular marker reversal. Following Cdk3 knockdown in SW620 cells (Figure 6D), we found that Cdk3 knockdown caused a mesenchymal to epithelial shift, including E-cadherin (Figure 6D-a) and α-catenin (Figure 6D-c) upregulation, and fibronectin (Figure 6D-e), snail (Figure 6D-g) and vimentin (Figure 6D-i) downregulation. Similarly, we also found by western-blotting that the expression of E-cadherin (Figure 6E, 2^nd^ panel) and α-catenin (Figure 6E, 3^rd^ panel) were increased, whereas vimentin (Figure 6E, 4^th^ panel), fibronectin (Figure 6E, 5^th^ panel), snail and vimentin (Figure 6E, 6^th^ panel) were decreased in SW620-siCdk3 cells. Simultaneously, AP-1 activity was significantly low in SW620-siCdk3 in comparison with SW620-simock (Figure 6F, lane 4 *vs*. 5, * *P* < 0.05). To further confirm whether the activated AP-1 by Cdk3 participates in EMT shift, AP-1 activity was inhibited with curcumin in HT29-Cdk3 cells, and then EMT shift markers were detected. After curcumin treatment, E-cadherin and α-catenin were increased (Figure 6G, right lane in 2^nd^, 3^rd^ panels), and fibronectin, snail and vimentin were decreased (Figure 6G, right lane in 4^th^, 5^th^,6^th^ panels). The shift from epithelial to mesenchymal was blocked when AP-1 activity being inhibited. These results indicate that Cdk3 activating AP-1 plays an important role in EMT-like marker shift.

### Cdk3 induces EMT-like cellular marker alteration in metastatic tumors of nude mice

**Figure 7:**
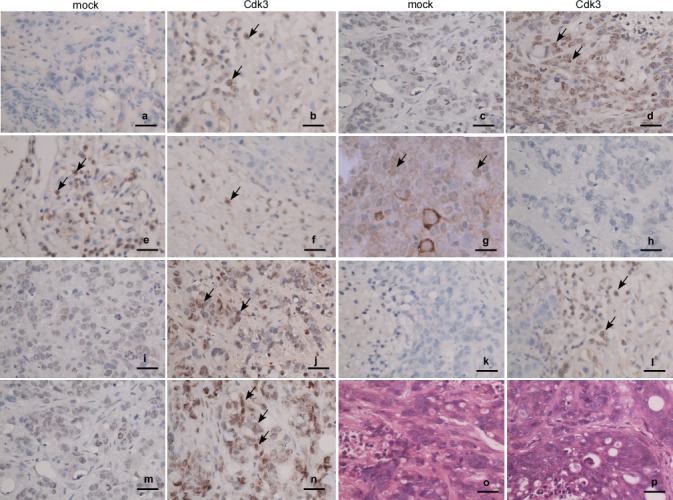
Expression of EMT markers in Cdk3-promoted metastatic tumors The metastatic tumors were subjected to paraffin embedding and section. Expressions of Cdk3, c-Jun, E-cadherin, α-catenin, vimentin, fibronectin and snail in the metastatic tumors were detected using immunohistochemistry assay. Paraffin sections were stained with antibody against Cdk3 (a, b), c-Jun (c, d), E-cadherin (e, f), α-catenin(g, h), fibronectin (i, j), vimentin (k, l) or snail (m, n), and also stained with hematoxylin and eosin (o, p). Arrows, positive cells. Magnification, ×40, scale bar = 10μm.

The above results showed that Cdk3 induces the EMT shift of colorectal cancer cell *in vitro*. To further confirm whether Cdk3-mediated EMT is involved in colorectal cancer metastasis *ex vivo*, the metastatic tumor samples of nude mice were used to test EMT-like marker expression. The immunohistochemistry staining revealed that the metastatic tissues of HT29-Cdk3 displayed E-cadherin (Figure 7-b) and α-catenin (Figure 7-d) downregulation, and fibronectin (Figure 7-f), vimentin (Figure 7-h) and snail (Figure 7-j) upregulation. This result consists with the above *vitro* data.

## DISCUSSION

Cdk3 functions in the regulation of G1-S cell cycle translation [17], it participates in G1-S progression by binding E2F1, E2F2, or E2F3. Recently, Cdk3 was found a novel function, which is involved in cancer development and progression. Cdk3 is overexpressed in a number of cancer tissues and cancer cell lines [21, 32, 33], and it has been found to be deregulated or mutated in numerous human tumors. Cdk3 is activated by DNA tumor virus proteins EIA, SV40 large T, and E7 through binding to pRb and consequent release of E2F [34]. Herpes simplex virus (HSV) can also activate Cdk3 [35]. Experimental studies showed that Cdk3 promotes cell transformation [15, 23, 24, 36]. Clinical investigations showed that Cdk3 highly expresses in various tumor [20–22], and its expression was associated with the degree of infiltration, lymph node metastasis and clinical staging [21, 22]. Ouelaa-Benslama R et al. reported that Cdk3 participates in EMT shift mediated by GαGβγ, AKT and PKCα [37]. Based on these, we speculated that Cdk3 may be involved in cancer metastasis. To confirm this speculation, we at first detected Cdk3 protein expression and activity in the biopsy tissues of colorectal cancer, and found that both Cdk3 expression and activity are high in metastatic tissues. Further, Cdk3 expression-vector was used to investigate its effect in cancer metastasis, we found that Cdk3 can promote the motility and invasion of colorectal cancer cell *in vitro* and metastasis *ex vivo*, mediate cell EMT shift. We think that Cdk3 is involved in not only cancer development but also metastasis.

To further analyze the mechanism of Cdk3-mediated metastasis, based on the previous finding, Cdk3 activating AP-1 through phosphorylating c-Jun, we investigated whether Cdk3-activating AP-1 participates in colorectal cancer metastasis. Our results showed that Cdk3 could phosphorylate c-Jun *in vitro* or *ex vivo*, and activate AP-1, increase cell motility and invasion. It is reported that as a major component of AP-1 [29], c-Jun phosphorylation plays an important role in AP-1 activation [24], and AP-1 activation though c-Jun phosphorylation participates in cell invasion [30, 31]. Cdk3 may directly be an upstream of c-Jun, and our data indicated that Cdk3 was a kinase involved in the signaling induced by EGF treatment. Phosphorylation of c-Jun at Ser63 and Ser73 mediates the transactivation function of c-Jun, which is required for its function as an oncoprotein. AP-1 activation occurs in various cancer [38] and plays an important role in cancer development and progression [39]. Based on the above, we think that Cdk3 increases AP-1 activity through c-Jun phosphorylation and promotes colorectal cancer metastasis.

Hepatic metastasis is the most common form of distant spread of primary colorectal cancer. It is estimated that approximately 50% of patients with colorectal cancer develop hepatic metastases synchro­nously or metachronously, and in advanced dis­ease the mortality of colorectal cancer is principally attributable to the development of hepatic metastases [1]. In this study, animal experiments showed that Cdk3-induced metastasis was observed in the livers and lungs of nude mice, and mainly existed in the livers, this consists with *Jemal A*'s reports [1]. The results suggested that Cdk3 plays an important role in hepatic metastases of colorectal cancer.

**Figure 8:**
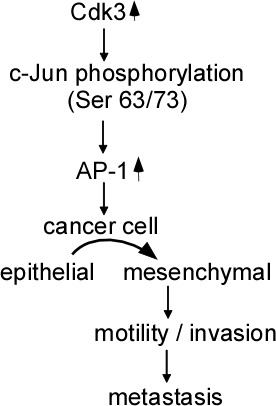
Schematic illustration of Cdk3-mediating colorectal cancer metastasis Cdk3 activates AP-1 through phosphorylating c-Jun Ser 63/73, mediates EMT alteration, increases motility and invasion, and promotes colorectal metastasis.

FoxM1 overexpression is significantly associated with colon cancer metastasis. FoxM1-mediated colon cancer metastasis is linked to regulating E-cadherin, vimentin, Snail expressions [13]. As a transcription factor of S100P, SOX9 binds to and activates S100P promoter, and induces invasiveness and metastasis of colon cancer cells. The decreased expression of SOX9 dramatically inhibits the tumor growth and peritoneal metastasis in nude mice. Furthermore, SOX9 regulates E-cadherin, N-cadherin, vimentin and Snail expressions at protein and mRNA levels, mediates EMT, promotes colon cancer metastasis [14]. We think that Cdk3 may also promote colorectal cancer metastasis through regulating EMT shift. The three reasons are illustrated as follows. The first one is that ectopic expressed Cdk3 caused an EMT-like marker shift, including a dramatic downregulation of the epithelial markers E-cadherin and α-catenin, and upregulation of the mesenchymal markers fibronectin and the EMT-associated transcription factor snail and vimentin. The second is that Cdk3 activated AP-1 through induction of c-Jun Ser 63/73 phosphorylation, AP-1 activation promoted cancer cell from epithelial to mesenchymal transition [25, 26]. And the third one is that EMT marker alternation existed in Cdk3-mediated metastatic tumor *ex vivo*. Taken together, our data indicated that Cdk3 activates AP-1through phosphorylating c-Jun Ser 63/73, induces EMT alteration, increases motility and invasion of colorectal cancer, and finally promotes colorectal cancer invasion and migration (Figure 8).

## MATERIALS AND METHODS

### Ethics statement

All animal works were conducted under the institutional guidelines of Hunan Province and approved by the Use Committee for Animal Care. Approval from the Zhuhai Hospital of Jinan University Ethics Committee and Xiangya Hopital of Central South University Ethics Committee was obtained, and written informed consent was provided by each human subject.

### Reagents and antibodies

Reagents including Tris, HCl, EDTA, KCl, NaCl, and SDS for buffer preparation, and curcumin were purchased from Sigma-Aldrich (St. Louis, MO). Cell culture medium and other supplements were obtained from Life Technologies (Rockville, MD). Cdk3 active kinase was purchased from Upstate Biotechnology, Inc. (Lake Placid, NY). Antibodies against Cdk3, E-cadherin, α-catenin, fibronectin, snail, and vimentin were from Santa Cruz Biotechnology, Inc. (Santa Cruz, CA) and Cell Signal Technology, Inc. (Beverly, MA). Taq DNA polymerase was purchased from Qiagen, Inc. (Valencia, CA). The jetPEI was purchased from Qbiogen, Inc. (Montreal, Quebec, Canada). G418 was from Biomol International, L.P. (Plymouth Meeting, PA). Matrigel was purchased from Collaborattiv Biomedical Products (Bedford, MA). Boden chamber was purchased from Neuro Probe (Cabin John, MD)

### Colon cancer sample

188 tissue specimens including 52 cases of normal colon tissues (NCE), 87 cases of primary colon cancer (PCC), and 49 cases of metastatic colon cancer (MCC) were obtained from Xiangya Hospital from Jan, 2007 to Sep, 2011. Every specimen was fixed with 40 g/L paraformaldehyde solution, followed by dehydration and paraffin embedment. 4 μm serial sections were utilized for immunohistochemistry. Additionally, for Cdk3 activity assay, the fresh tissues samples of 21 cases NCE, 27 cases PCC, and 24 cases MCC were obtained by surgical operation. All patients had only one primer tumor, and no one had received another treatment.

### Cell culture and treatments

HEK293, HT29, SW620, HCT116, SW480 and HCoEpiC (human colonic epithelia cell) were from Cell Center of Central South University (Changsha, CN). These cell lines were cultured with 1640 medium supplemented with 10% fetal bovine serum (FBS) and antibiotics at 37°C in a 5% CO2 incubator. Colorectal cancer cell lines, HT29 and SW480 have a low metastatic ability, SW620 and HCT116 have high ability [40]. The cells were maintained by splitting at 90% confluence and media were changed every 3 days. For transfection experiments, cells at 50 to 60% confluence were transfected using jetPEI (Qbiogen, Inc.) following the manufacturer's suggested protocol. HCoEpiC cells were transfected with pRcCMV-HA-Cdk3 and pHis6-tagged c-Jun for determine the binding of Cdk3 and c-Jun. HT29 cells were transfected with pRcCMV-HA-Cdk3 or pRcCMV-HA (mock). Cdk3 stably-transfected cell line and mock cell were obtained by selection for G418 resistance (400 μg/ml), and further confirmed by assessing Cdk3 activity and expression [41]. For Cdk3 rescue experiments, SW620-siCdk3 cells were transiently transfected with pRcCMV-HA-Cdk3, and then used for motility and invasion assay, and Cdk3 expression and activity assay. TH29-Cdk3 cells were treated with curcumin at 10 μM for 30 min for motility and invasion assay. The treated cells were used to detect E-cadherin, α-catenin, fibronectin, vimentin and snail expression, and these treated cells were also used for AP-1 activity assay [42].

### Immunohistochemistry

Immunohistochemistry was done on the formalin-fixed and paraffin-embedded tissue sections using a standard immunohistochemical technique. 4μm thick tissue sections were deparaffinized in xylene, rehydrated in a graded alcohol series, and treated with an antigen retrieval solution (10 mmol/L sodium citrate buffer, pH 6.0) [43]. The sections were incubated with anti-Cdk3, c-Jun, E-cadherin, α-catenin, fibronectin, vimentin or snail antibody (Abcam, dilution 1:50) overnight at 4°C. Subsequently, the sections were incubated with a biotinylated secondary antibody (Zhongshan Inc., China), followed by incubation with an avidin–biotin complex (Zhongshan Inc., China) according to the manufacturer's instructions. Finally, tissue sections were incubated with 3′,3′-diaminobenzidine (DAB) (Sigma-Aldrich) and hydrogen peroxide for 2 min, and counterstained with haematoxylin for 30 s. In negative controls, primary antibodies were omitted.

### Evaluation of Cdk3 staining

Sections were blindly evaluated by two investigators in an effort to provide a consensus on staining patterns under light microscopy (Olympus). Cdk3 staining was assessed according to the methods described by Cheng AL [44] and Li YJ [45] with minor modifications. Each case was rated according to a score that added a scale of intensity of staining to the area of staining. At least 10 high-power fields were chosen randomly, and >1000 cells were counted for each section. The depth of staining was graded on the following scale: 0, no cell coloration; 1+, light yellow; 2+, brown; 3+, tan. The area of staining was evaluated as follows: 0, no staining of cells in any microscopic fields; 1+, < 30% of tissue stained positive; 2+, between 30% and 60% stained positive; 3+, >60% stained positive. The summed (extension + intensity) was used as the total score, where 0–1 indicates a negative score (−); ≥2 a positive score (+) [45]. The correlation of CDK3 expression with clinical features and prognosis of colorectal cancer patients was analyzed. Statistical analysis was done using SPSS (version 18.0), a difference of P < 0.05 was considered statistically significant.

### Cell motility and invasion assay

For cell invasion assay, the treated cells were removed by trypsinization, and their invasiveness was tested by the Boden chamber invasion assay *in vitro* [46]. Matrigel (Collaborattiv Biomedical Products, Bedford, MA) was diluted to 25 mg/50 ml with cold filtered distilled water and applied to 8 mm pore size polycarbonate membrane filters. The cells were seeded to Boden chamber (Neuro Probe, cabin John, MD) at the upper part at a density of 1.5 × 10^4^ cells/well in 50 μl of serum-free-medium, and then incubated for 12 h at 37°C. The bottom chamber also contained standard medium with 20 % fetal bovine serum (FBS). The cells invaded to lower surface of membrane were fixed with methanol and stained with hematoxylin and eosin. Random field was counted for invaded cells under a light microscope. To determine cell motility, the cells were seed into Boyden Champer on membrane filters, which not coated with Matrigel. Migration of cells was measured as described in the motility assay [46]. Statistical analysis was corrected with cell viability to clarify the effect of Cdk3

### Construction of expression vectors

pRcCMV-HA-Cdk3, pRcCMV-HA were gifts from Dr. Barrett J. Rollins (Harvard Medical School, Boston, Massachusetts) [17]. pU6pro vector was provided by David L. Turner (University of Michigan, Ann Arbor, MI). pU6pro was used to construct pU6pro-simock (simock) and Pu6pro-siCdk3 (siCdk3) following the recommended protocol. For constructing the simock and siCdk3, we synthesized primers for simock (general scramble: sense TTCAAGAGACACCTATAACAACGGTAGTTTTTT-3′, and antisense ACTACCGTTGTTATAGGTGTCTCTTGAACACCTAT AACAACGGTAGT-3′). siCdk3 was synthesized using primers (sense5′-TTTGTGAGTTGGGTGCCATCAAGTTCAAGAGACTTGATGGCACCCAACTCATTTTT-3′, and antisense AAAATGAGTTGGGTGCCATCAAGTCTCTTGAACTT GATGGCACCCAACTCA-3′). All constructs were confirmed by restriction enzyme mapping and DNA sequencing [24]. AP-1 luciferase reporter plasmid contains the −73 to +63 collagenase promoter sequence [47]. CMV-neo marker vector plasmid was constructed as previously reported [47, 48]. pHis6-tagged c-Jun was kindly provided by Dr. Dirk Bohmann (European Molecular Biology Laboratory, Heidelberg, Germany) [49]. c-Jun fragment was generated by PCR and subcloned into the pGEX-5x-1 vector (Amersham Biosciences Corp., Piscataway, NJ) at the *BamHI/Xhol* site to generate a glutathione S-transferase (GST)-c-Jun plasmid (pGST-c-Jun) [47, 50].

### Western-blotting

Equal numbers (1.0 ×10^6^) of the treated cells were harvested, and washed once with ice-cold phosphate-buffered saline (PBS). The cell samples were disrupted with 0.6 ml RIPA buffer (1× PBS, 1% Nonidet P-40, 0.5% sodium deoxycholate, 0.1% SDS, and freshly added 100 μg/ml phenylmethanesulfonyl fluoride, 10 μg/ml aprotinin, 1 mM sodium orthovanadate). The cell lysates were clarified by microcentrifuge and the supernatant fractions were saved to perform next steps. The concentration of supernatant fractions was determined using the Bio-Rad Protein Assay (Bio-Rad Laboratories, Inc., Hercules, CA). The samples containing equal amounts of protein in an equal volume of RIPA buffer were diluted with 5×SDS sample buffer [187.5 mM Tris-HCl pH 6.8, 6% (w/v) SDS, 30% (v/v) glycerol 150 mM dithiothreitol (DTT) and 0.3% (w/v) bromophenol blue]. Then samples were separated by 10% SDS-PAGE, and proteins were transferred on Nitrocellulose membrane [51, 52]. The membrane was subsequently incubated with 5 % non-fat milk in PBS for 1 h to block non-specific binding, and incubated with specific antibody for 2 h, and then with an appreciate peroxidase-conjugated secondary antibody for 1 h. All incubations were carried out at 37°C, and intensive PBS washing was performed each incubation. After 3 times PBS washing, signal was developed by 4-chloro-1-napthol/3,3-o-diamino-benzidine, and relative photographic density was quantitated by a gel documentation and analysis system. β-actin was used as an internal control to verify basal level expression and equal protein loading. Abundance ratio to β-actin was counted.

### Cdk3 immunoprecipitation kinase assay

For Cdk3 activity assay of tissue samples, eight-micrometer-thick frozen sections of fresh NCE, PCC, and MCC were prepared using a Leica CM 1900 cryostat (Leica) at −25°C. The sections were placed on a membrane-coated glass slides (2.0 μm, 50 pieces, PEF Membrane; Leica), fixed in 75% alcohol for 30 s, and stained with 0.5% violet-free methyl green (Sigma). The pooled microdissected cells from 21 NCE, 27 PCC, or 24 MCC specimens were used for *vitro* kinase assay. Each cell population was determined to be 95% homogeneous by microscopic visualization of the captured cells [53, 54]. The microdissected cells were dissolved in kinase lysis buffer [25 mM Tris-HCl (pH 7.5), 5mM β-glycerophosphate, 0.1 mM Na3 VO4, 10 mM MgCl2, 1 mM aprotinin and 1 mM PMSF] at 4°C for 1 h, and then centrifuged. Protein concentration of the clarified supernatant fractions was measured. 100 μg protein of the supernatant fraction was subjected to immunoprecipitation using Cdk3 antibody. The immune complexes were used to *in vitro* kinase assay. All solutions for staining were supplemented with protease inhibitor cocktail tablets (Roche Molecular Biochemicals).

For cell Cdk3 activity assay, the treated cells (1.0×10^6^) were cultured for 12-24 h in 100-mm dishes. After 70-80% confluence, the cells were washed once with ice-cold PBS, then harvested and disrupted in 250 μl of kinase lysis buffer. The lysates were centrifuged. The protein concentration of supernatant fraction was measured. The supernatant fractions were subjected to immunoprecipitation using Cdk3 antibody. The immune complexes were used to *in vitro* kinase assay.

Cdk3 kinase assay of immunoprecipitation was carried out as described by the protocol from Upstate Biotechnology, Inc (Upstate Biotechnology, Inc., Lake Placid, NY). Briefly, the immune complex was added to 2.5μl of 10 × kinase buffer [250 mM Tris-HCl (pH 7.5), 50mM β-glycerophosphate, 20 mM DTT, 1 mM Na_3_ VO_4_, 100 mM MgCl_2_], 2.5 μl (2.5 μg) of GST-c-Jun protein, 10 μl diluted ATP/cocktail (Upstate Biotechnology, Inc.), 10 Ci of [γ^32^] ATP and H_2_O added to 25μl. The reaction was incubated at 30°C for 30 min and then subjected to separation by 12% SDS-PAGE. c-Jun was analyzed by autoradiography as described [52, 55].

### *In vitro* kinase assay

To determine phosphorylation of c-Jun by active Cdk3, a Cdk3 kinase assay was carried out at 30°C for 30 min in the presence of the kinase buffer [25 mM Tris-HCl (pH 7.5), 5mM β-glycerophosphate, 2 mM DTT, 0.1 mM Na_3_ VO_4_, and 10 mM MgCl_2_] with 200 μM ATP and 2 μg of GST-c-Jun as substrate. The reaction solution was subjected to Western-blotting. The phosphorylation of c-Jun was analyzed by Western-blotting using antibody against c-Jun Ser63 or Ser73 as described [55].

### Immunofluorescence assay

The treated cells (1.0×10^3^) were seeded in eight-chamber slides and incubated 24 h at 37°C, 5% CO_2_, washed at each time point, fixed in 4% paraformaldehyde. The cells were hybridized with mouse monoclonal antibody against E-cadherin, α-catenin, fibronectin, snail, vimentin or mouse at room temperature for 4 h. After being washed, the cells were hybridized with anti-rabbit goat antibody conjugated with Texas Red or antibody against mouse conjugated with DAPAI for detection of cell nuclear. For determining co-localization of Cdk3 and c-Jun, HT29-Cdk3 and HT29-mock cells were hybridized with c-Jun mouse monoclonal antibody and Cdk3 rabbit monoclonal antibody for 4 h. After the first hybridization, the cells were washed and then hybridized with anti-mouse goat antibody conjugated with Texas Red for c-Jun detection or anti-rabbit goat antibody conjugated with FITC for Cdk3. After the second hybridization, the cells were washed and visualized with a fluorescence microscope (×400) [56].

### Reporter gene assays

Reporter gene assay for firefly luciferase activity was performed using lysates from transfected cells. The reporter gene vector pRL-SV40 (Promega, Madison, WI) was co-transfected into each cell line, and *Renilla* luciferase activity generated by this vector was used to normalize the results for transfection efficiency. Cell lysates were prepared by first washing the transfected HEK 293 cells or Cdk3 stably-transfected cells once in PBS at room temperature. After removing PBS completely, 100 μl lysis buffer (Promega Dual Luciferase Reporter Assay System) was added. Then cells were incubated for 1 h with gentle shaking. The lysates were then transferred into a reaction tube, and the cellular debris was removed by centrifugation. The supernatant fraction was used for the measurement of firefly and *Renilla* luciferase activity. 20 μl cell lysates were mixed with 100 μl Luciferase Assay II reagent, and firefly luciferase light emission was measured by Luminoskan Ascent plate reader (Thermo Electron Corp., Helsinki, Finland). Subsequently, coelenterazine reagent (100 μl) containing the substrate for *Renilla* luciferase light emission was mixed to normalize the firefly luciferase data. The results were expressed as relative AP-1 activity (fold or percentage) as described previously [57, 58].

### Animal

A total of 20 female nude BABL/c mice (approximately 5 - 6 weeks old) were purchased from Animal Center of Central South University. They were maintained in the Laboratory for Experiments, Central South University under laminar airflow conditions. The studies were conducted according to the standards established by the guidelines of Guangdong Province and approved by the Use Committee for Animal Care [43].

### Colon cancer metastasis in nude mice

The metastatic effect of Cdk3 expression on HT29 cell *ex vivo* was determined as described previously with some modifications [59]. Briefly, 100 μl aliquots of HT29-Cdk3 or TH29-mock suspensions (1×10^4^ cells) were mixed with Matrigel, and then respectively injected into the tail veins of nude mice (10 mice per group). These mice were bred for 60 days. After 60 days, the mice were sacrificed, and the metastasis was observed in lung, liver and lymph nodes [43, 60]. Metastasis was evaluated by measuring weight of metastasized tumors at the lung and liver.

## SUPPLEMENTARY MATERIAL FIGURES AND TABLE


