# Supplementary material for: Cdk3-promoted epithelial-mesenchymal transition through activating AP-1 is involved in colorectal cancer metastasis
Source: Oncotarget. 2016 Jan 9;7(6):7012–28. doi: 10.18632/oncotarget.6875 (PMC4872765; doi:10.18632/oncotarget.6875)
Supplement: Supplementary file 1 [file oncotarget-07-7012-s001.pdf]

Cdk3-promoted epithelial-mesenchymal transition through activating AP-1 is involved in colorectal cancer metastasis

Supplementary Material

Supplemental Table Analysis of relationships between Cdk3 expression and clinicopathological characteristics of colorectal cancer patients

Variables	Number	Cdk3 (score) Median (min–max)	*P* value
Gender			
Men	82	12.4(4.4-25.6)	0.823
Women	54	12.0(8.0-19.0)1	
Age, years			
≤45	64	11.9(4.4-25.6)	0.855
>45	72	12.5(7.8-19.0)	
Smoking			
Yes	91	12.4(6.5-25.6)	0.583
No	45	12.5(4.4-19.0)	
Alcohol intake			
Yes	89	12.5(5.0-25.6)	0.675
No	47	12.4(4.4-17.5)	
TNM grade			
I+II	52	9.2(4.4-17.3)	0.0001
III+IV	84	13.1(8.9-25.6)	

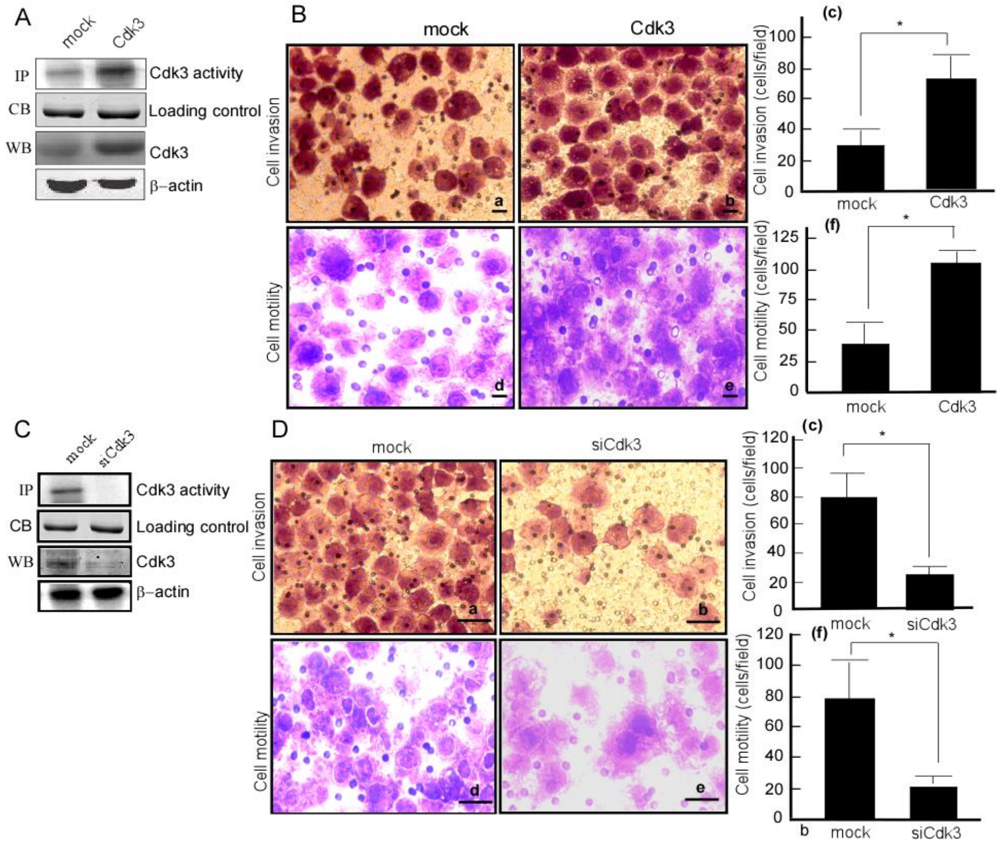

Supplemental Figure 1. ***Cdk3 increases*** *SW480* ***motility and invasion and siCdk3 decreases HCT116 motility and invasion.*** A, Cdk3 expression was detected in SW480-Cdk3 and SW480-mock using Western-blotting. Cdk3 activity was detected using immunoprecipiptation and *in vitro* kinase assay. B. Matrigel-coated Boyden chamber was used to measure the invasion of SW480-mock (a) and SW480-Cdk3 (b). The invaded cells were counted (c). The uncoated Boyden chamber was used to determine these cells motility (d, e), and the motility cells were counted (f). C. Cdk3 expression and activity were detected in HCT116-simock and HCT-siCdk3. D. The invasion of HCT116-simock (a) and HCT116-siCdk3 (b) was detected, and the invaded cells were counted (c). The motility of HCT116-simock (d) and HCT116-siCdk3 (e) was detected. The invaded cells were counted (f). Original magnification, ×400. Scale bar = 20μm. The cells were counted in three individual experiments and presented as mean ± SD. *, represented *P* <

0.05. Coommassie blue staining and β-actin served as a loading control. IP, immunoprecipiptation assay; CB, Coommassie blue staining; WB, Western-blotting analysis. *, represented $P < 0.05$.

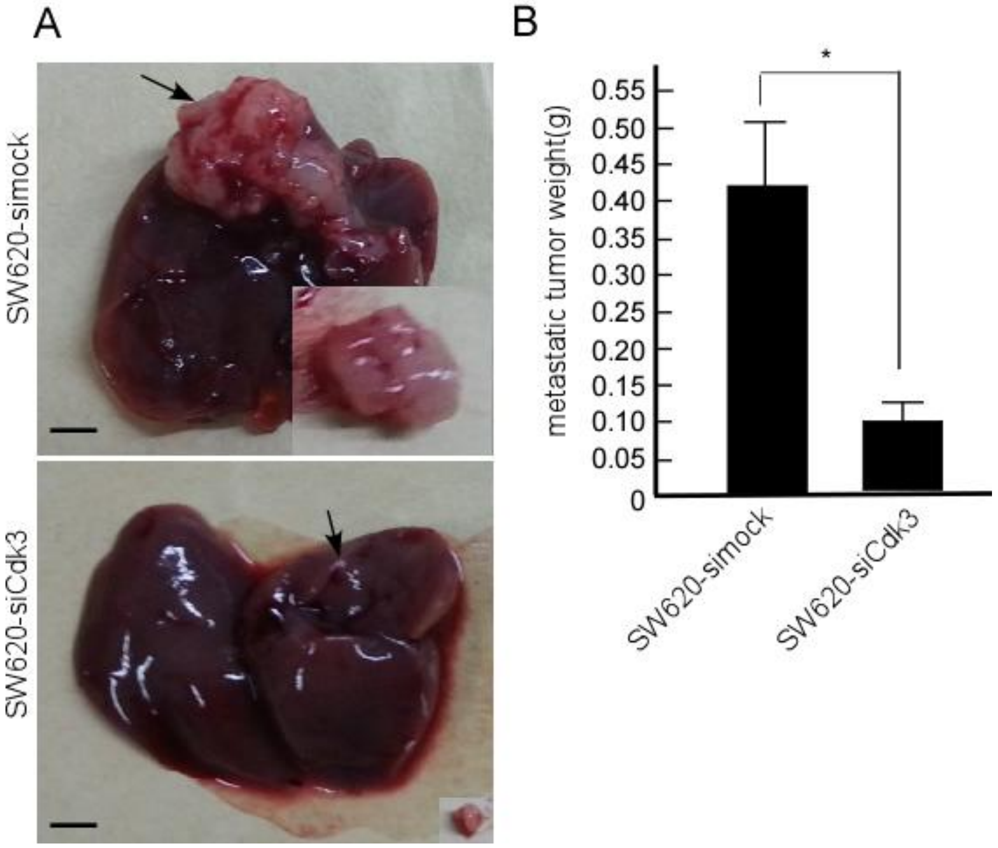

Supplemental Figure 2. ***siCdk3 decreases colorectal cancer cell metastasis ex vivo.*** A, 20 nude mice were randomly divided into two groups with 10 mice per group. One group was injected with SW620-siCdk3 cells in Matrigel through the tail vein, and the other group was injected with SW620-simock as described in Material and methods. The metastatic tumors were observed in lung, liver and lymph nodes. Arrows, metastatic node. B. the metastatic tumors from the lung and live were weighed. Scale bar = 0.5 cm (*, $p < 0.05$).
